# Neurological Screening in Elderly Liver Transplantation Candidates: A Single Center Experience

**DOI:** 10.3390/neurolint14010019

**Published:** 2022-02-25

**Authors:** Federica Avorio, Gianvincenzo Sparacia, Giovanna Russelli, Aurelio Seidita, Giuseppe Mamone, Rossella Alduino, Fabio Tuzzolino, Salvatore Gruttadauria, Roberto Miraglia, Matteo Bulati, Vincenzina Lo Re

**Affiliations:** 1 Neurology Service, Mediterranean Institute for Transplantation and Advanced Specialized Therapies (IRCCS-ISMETT), Via Ernesto Tricomi 5, 90127 Palermo, Italy; favorio@ismett.edu; 2 Radiology Service, Department of Diagnostic and Therapeutic Services, Mediterranean Institute for Transplantation and Advanced Specialized Therapies (IRCCS-ISMETT), Via Ernesto Tricomi 5, 90127 Palermo, Italy; gsparacia@ismett.edu (G.S.); gmamone@ismett.edu (G.M.); rmiraglia@ismett.edu (R.M.); 3 Radiology Service, Department of Biomedicine, Neurosciences and Advanced Diagnostics (BiND), University of Palermo, 90100 Palermo, Italy; 4 Research Department, Mediterranean Institute for Transplantation and Advanced Specialized Therapies (IRCCS-ISMETT), Via Ernesto Tricomi 5, 90127 Palermo, Italy; grusselli@ismett.edu (G.R.); ralduino@ismett.edu (R.A.); ftuzzolino@ismett.edu (F.T.); mbulati@ismett.edu (M.B.); 5 Department for the Treatment and Study of Abdominal Diseases and Abdominal Transplantation, Mediterranean Institute for Transplantation and Advanced Specialized Therapies (IRCCS-ISMETT), Via Ernesto Tricomi 5, 90127 Palermo, Italy; aseidita@ismett.edu (A.S.); sgruttadauria@ismett.edu (S.G.); 6 Department of Surgery and Surgical and Medical Specialties, University of Catania, 95124 Catania, Italy

**Keywords:** cerebral small vessel disease, liver transplantation, brain magnetic resonance imaging elderly age-related diseases

## Abstract

Background: Cerebral small vessels disease (cSVD) is an age-related disorder and risk factor for stroke and cognitive/motor impairments. Neurological complications (NCs) are among the causes of adverse outcomes in older liver transplant recipients. This study sought to determine whether cSVD predicts acute NCs in over 65-year-old liver transplant patients. Methods: Data were collected, from a retrospective medical chart review, of 22 deceased donor liver transplant recipients aged 65 years or older with a pre-operative brain magnetic resonance imaging (MRI). We used the Fazekas score (0–3) as a quantitative measurement of the vascular lesion load seen in the MRI. We analyzed all post-operative acute NCs occurring during the hospital stay and any other non-NC. Results: cSVD was recognized in all patients. Neurological complications (NCs) occurred in 18.1% of patients with toxic-metabolic encephalopathy the most frequent diagnosis (13.64%). More severe cSVD was associated with seizures (*p* = 0.0362), longer hospital stay (*p* 0.0299), and disability (*p* 0.0134). In our elderly cohort, hepatic encephalopathy (HE) (*p* 0.0287) and ascites (*p* 0.0270) were predictors of NCs after liver transplantation. Ascites and/or variceal bleeding and severity of liver disease were associated with adverse post-operative outcomes. The small sample size limited the statistical analysis power. Conclusions: We present the preliminary data of a single-center retrospective study aimed at understanding the cSVD role on NCs and non-NCs after a liver transplantation in elderly patients. This would encourage a more appropriate multicenter prospective study that will definitely confirm if a neurological screening in old age liver transplant candidates is appropriate.

## 1. Introduction

The increase in life expectancy and improvements in medical management have led to an increased number of elderly patients affected with chronic hepatic disease, and their placement on liver transplant waiting lists has become more frequent. Similarly, the number of elderly liver recipients has increased: in 1988, only 29 patients ≥ 65 years old underwent a liver transplant in the United States; in 2005, these patients were 628 in number, with an increase of 2100% [1].

The favorable outcomes of liver transplants in patients over 60 years old, reported in the early 1990s by individual transplant centers, made old age a non-contraindication to transplantation. In 1991, the University of Pittsburgh also reported a similar three year survival between elderly and young patient populations (65.5% and 71.4%, respectively) [2]. Differently from previous reports of individual centers reporting similar survival rates in young and elderly liver transplant recipients, in 1998, a methodologically more appropriate multicenter review of 735 liver transplant recipients, analyzed on the basis of age, showed a reduced survival for over-60-year-old recipients compared to young recipients (81% vs. 90%; *p* = 0.04). Increased mortality in elderly patients was primarily not due to non-hepatic causes, but to neurological causes, in addition to cardiac and infectious causes, within 6 months after surgery [3]. Nevertheless, risk factors associated with this increased mortality have not been identified, and the authors recommend not considering age as a factor that precludes the transplant, but to perform a more aggressive and extended screening of age-related comorbidities when assessing elderly patients affected with a chronic liver disease and potential liver transplant recipients.

Elderly patients carry higher risk of being affected with age-related comorbidities [4], and therefore the pre-transplant clinical evaluation is more extensive and should include a screening of age-related diseases that can potentially affect the post-transplant outcomes. The pre-transplant clinical assessment, to establish the eligibility of elderly cirrhotic patients for a liver transplant, differs among centers and is not standardized. Most centers include a screening for coronary artery disease, bone diseases (osteopenia or osteoporosis), and tumors, while the screening for cerebral small vessel disease (cSVD), one of the most common neurological diseases in patients aged over 60, is usually not performed as a standard.

In the last two years, chronic cerebrovascular disease characterized by microangiopathy has been recognized as a serious problem for the general elderly population and, even in subclinical forms, causes cognitive, psychiatric, and motor impairments. It is also responsible for one-fifth of all strokes, it doubles the risk of future strokes, and contributes up to 45% of all dementias. It is therefore understandable how high its cost is for the health system [5]. The typical radiological markers are white matter lesions (WML) with variable load and size, mainly distributed in the subcortical and periventricular white matter. At the beginning, these are isolated lesions that later merge, reaching a severe picture of leukoaraiosis. Other radiological/pathological markers include microbleeds, dilated Virchow–Robin spaces, lacunes, and cortical atrophy. Because age is the main risk factor for such neurological conditions, one of its characteristics is a tendency to constantly progress over time, similarly to neurodegenerative diseases [6]. The broad clinical variability depends on the volume and site of WML, and probably also on other factors such as the individual’s cognitive reservoir [7].

Consistent with the previously reported conclusions [3], in our transplantation center, we included cSVD screening in the pre-transplant assessment of patients ≥ 65 year old to understand whether this age-related comorbidity is a risk factor for post-operative adverse outcomes, focusing mainly on acute neurological complications, those reported among main causes of mortality in elderly liver transplant recipients.

## 2. Materials and Methods

### 2.1. Study Design

We conducted a retrospective cohort study in a single transplantation center with the aim of verifying the impact of chronic cerebrovascular disease on the risk of post-operative adverse neurological and non-neurological outcomes in liver transplant recipients ≥ 65 year old. The study was approved by the Institutional Research Review Board (IRRB/22/20) and the Ethics Committee (EC).

### 2.2. Patient Population

The cohort was extracted from a sample of patients ≥ 65 years old suffering from cirrhosis and who underwent a deceased donor liver transplant between June 2014 and February 2021. The selected cohort included all consecutive patients with at least a brain magnetic resonance imaging (MRI) conducted in the pre-transplant assessment. The median number of days from brain MRI to transplant was 72.5 days (22-1849). We also included only those patients who previously provided a consent to use their personal data for research purposes.

### 2.3. Description of Clinical Assessments and Protocols

The liver disease was assessed by an expert hepatologist, and the severity of the liver disease was classified according to the Model for End-Stage Liver Disease (MELD) score. A neurology consultant, with expertise in neurological complications of solid organ transplants and end-stage diseases, carried out the pre- and post-transplant neurological assessment. The pre-operative clinical assessment was made to verify the presence or absence of clinical manifestations related to a chronic cerebrovascular disease, such as cognitive impairment (although a definite etiology is difficult to establish in liver disease patients who also have a metabolic-type subcortical brain injury) and motor disorders. The post-operative neurological assessment was aimed at identifying acute neurological complications after surgery. This assessment was both clinical and instrumental (neurophysiology and neuroimaging techniques) when deemed necessary. Immunosuppressant regimen consisted of 20 mg of intravenous basiliximab on day 0 and on postoperative day (POD) 4, followed by tacrolimus-based immunosuppression from POD 1 (0.05–0.1 mg/Kg) with a target blood level of 8–10 ng/mL until to POD 90. The 12 h trough concentrations of tacrolimus (IMx whole-blood assay, Abbott Laboratories, Abbott Park, Lake Bluff, IL, USA) were analyzed daily for the first week after transplantation. Neuroimaging protocol: all brain MRI scans were acquired with a 1.5 (Signa Explorer, GE Healthcare) or 3.0 (Discovery 750w, GE Healthcare) Tesla scanner, following a standard protocol that included T1- and T2-weighted axial sequences and fluid-attenuated inversion recovery (FLAIR) (5 mm thickness, 1 mm interval) and T2* gradient-echo (GRE) or susceptibility-weighted imaging (SWI) sequences. Images were assessed by an expert neuroradiologist blinded to the patient’s clinical course.

### 2.4. Observation Parameters

All variables were obtained by reviewing the medical records: (1) demographic information: age, gender, body mass index (BMI); (2) pre-operative clinical characteristics: vascular risk factors (hypertension, smoking, hyperlipidemia, diabetes mellitus, atrial fibrillation, history of stroke/transient ischemic attack (TIA), kidney failure), etiology of liver disease with severity (Model for End-Stage Liver Disease (MELD) and United Network for Organ Sharing (UNOS) status) and complications, presence of transjugular intrahepatic portosystemic shunt (TIPS), and waiting list time; (3) donor variables: age and gender; (4) intra-operative variables: operation time, cold and warm ischemia time; (5) peri-operative laboratory variables: sodium shift (positive if we registered a shift level ≥8 mEq/L/day [8] from the admission to POD 7), tacrolimus (a calcineurin inhibitor with neurotoxic potentiality), and magnesium and sodium serum levels on POD 7; (4) radiological characteristics: type, number, size, and distribution of subcortical and periventricular WML were assessed and quantified with a semi-quantitative rating scale: Fazekas scale (0–3) [9]. We looked at post-operative acute neurological complications (NCs) and non-neurological complications (respiratory, renal, infective, rejection, graft failure) as reported in the clinical course description. In the case of more than one non-neurological complication, we considered the most severe one. General outcomes were also registered: length of stay (LOS), length of intensive care unit (ICU) stay, and discharge modality (home without assistance vs. home with physical therapy or rehab facility) as a patient’s autonomy surrogate and in-hospital mortality.

### 2.5. Statistical Analyses

Continuous and categorical variables are expressed as median with interquartile range, and as frequency with percentage, respectively. To compare continuous variables, we used Wilcoxon tests or *t*-test when appropriate. Fisher’s exact tests were used to compare categorical variables among groups. To explore the differences between the recipients’ pre-operative risk factors in predicting events (peri-operative acute neurological complications and non-neurological complications, including more general outcomes), we used the chi-squared test, Fisher’s exact test, and logistic regression models or generalized linear models when appropriate. Levels of significance were set with a *p* < 0.05. Statistical analyses were performed using SAS software version 9.4.

## 3. Results

From June 2014 to February 2021, 51 cirrhotic patients ≥ 65 year old were transplanted at our institute. Only 22 of 51 were enrolled according to the inclusion criteria (pre-operative brain MRI and consent form signed). The demographic and clinical data of the selected cohort are summarized in Table 1. Patients’ average age at transplantation was 68 years old, with a range between 65- and 71-years-old. More precisely, 54.55% (12/22) of the recipients were between 65 and 67 years, 40.9% (9/22) between 68 and 70 years, and 9% (2/22) > 70 year old.

All patients had a Fazekas score > 0; in more detail, 11/22 (50%) had a score = 1, 8/22 (36.36%) had a Fazekas score = 2, 3/22 (13.64%) had a more severe cSVD with score = 3. The comorbidity cSVD was subclinical in all transplant candidates; we observed no major motor impairment. The cognitive assessment screening was over the cut-off for dementia suspicion, and for patients with a Mini Mental State Examination (MMSE) score < 30, the cognitive impairment was limited to attentive function items as expected in covert hepatic encephalopathy (HE). The observation period was limited to the perioperative surgical hospital stay. We registered a peri-operative sodium shift (greater than 8 mEq/L) in 36.4% of the enrolled patients; the average of tacrolimus level on POD 7 was 7.66 (reference range: 8–12 ng/mL). The in-hospital mortality was 0. The median LOS was 16.5 days (interquartile range [IQR]: 14–30), and the median length of ICU stay was 6 days (IQR: 3–7). In cases of ICU readmission (3), we considered the total number of ICU days; 19 out of 22 (86.36%) patients were discharged home without assistance, while 2/22 (9.09%) were discharged home with physical therapy prescription, and 1/22 (4.54%) was transferred to a rehab facility.

### 3.1. Influence of Different Pre/Peri-Transplant Factors on Postoperative Neurological and Non-Neurological Outcomes

Postoperative acute NCs in ≥65 old liver transplanted recipients are reported in Table 2; 4/22 (18.1%) of patients experienced at least one acute NC after surgery, and 2/22 (9.09%) presented more than one neurological diagnosis: toxic metabolic encephalopathy and central pontine myelinolysis (CPM), in one case, and simultaneously with pharmacological-resistant seizures in another case. The most frequent NC was toxic-metabolic encephalopathy (3/22, 13.64%), followed by CPM (2/22, 9.09%) and seizures (1/22, 4.55%).

We reported a regular course in 27.27% (6/22) of cases. The others were mainly pluri-complicated cases. All non-NCs are listed in Table 3.

The comparison between patients with NCs and patients without NCs are shown in Table 4. Both groups were similar in almost all demographic, clinical, and laboratory recipient variables, as well as donor and surgery variables.

We found a statistically significant association between Fazekas 3 score and seizures (*p* = 0.0362) (Table 5), while renal failure and perioperative sodium shift were independent predictors of toxic-metabolic encephalopathy (*p* = 0.0348 and *p* = 0.0497, respectively) and CPM (*p* = 0.0348 and *p* = 0.0497, respectively). Lower mean values of BMI (<22 Kg/m^2^) were associated with higher risk of toxic-metabolic encephalopathy and CPM (both *p* = 0.0456). Considering all NCs globally, we found a statistically significant association with preoperative HE (*p* = 0.0287) as well as ascites (*p* = 0.0270). Patients with acute NCs were more likely to have a longer ICU stay (*p* = 0.0064) and overall length of hospital stay (*p* = 0.0072).

Donor age (older) was associated with acute rejection (*p = 0.0444*). We also found an association between ascites and sodium POD7 values with acute renal impairment (*p = 0.0270* and *p = 0.0229*).

### 3.2. Influence of Different Pre/Peri-Transplant Factors on General Outcomes

Severe cSVD (Fazekas 3) was significantly associated with longer hospital stay (*p* = 0.0299), as were HE (*p* = 0.0092), ascites (*p* = 0.0066), variceal bleeding (*p* = 0.0024), and liver diagnosis (HCV in cirrhosis) (*p* = 0.0143). When peri-operative sodium shift occurred, this increased the risk of a longer LOS (*p* = 0.0004).

After analyzing continuous variables, we found the following associations with overall LOS stay: higher MELD score (*p* = 0.0147), longer operation time (*p* = 0.0035), and higher FK POD1 levels (*p* = 0.0390). When ICU stay was evaluated, we found similar associations: MELD score (*p* = 0.0040), HE (*p* = 0.0019), ascites (*p* = 0.0043), variceal bleeding (*p* = 0.0378), operation time (*p* = 0.0240), sodium shift (*p* = 0.0049), and magnesium POD7 (*p* = 0.0337).

HE and Fazekas 3 were associated with non-autonomous discharge/disability (*p* = 0.00412 and *p* = 0.0134, respectively).

### 3.3. Study Limitations

The main limitation of this study was the small number of patients analyzed, which did not guarantee that the statistical method primarily used (e.g., chi-squared test or Student’s *t*-test) were confirmed after applying a more robust statistical method for analysis (Fisher’s exact test and logistic regression models).

## 4. Discussion

Neurological complications are reported among the main causes of averse outcomes after liver transplantation in elderly recipients, raising the need to more extensively explore risk factors as well as age-related comorbidities. It is well known that NCs are in general related to pre-operative hepatic encephalopathy, poor nutritional status, and electrolyte imbalances [10]. Our group previously reported acute cerebrovascular disease as the most common neurological complication in liver transplanted patients [11]. Age-related cerebral small vessel disease has been recognized as a strong predictor of future stroke in the general population, as well as an independent risk factor for non-focal neurological injuries (encephalopathy and generalized seizures) after aortic arch repair surgery [12]. For all these reasons, we believe that age-related neurological comorbidity cSVD is worth being screened before liver transplantation in the elderly.

The incidence of neurological complications in our elderly cohort (18%) was similar to that expected from existing literature data [13]; therefore, in our small group, we did not record a higher risk of postoperative neurological adverse outcomes despite older age. The NC diagnoses we found were also congruent with the spectrum of neurological disorders thus far reported in the literature, confirming encephalopathy (13.64%) as the most frequent acute neurological complication, mainly related to neurotoxicity of immunosuppressant drugs and systemic metabolic derangements.

Although not confirmed by stronger statistical methods for the small sample size, the cSVD severity, particularly a Fazekas score of 3, was found to be a predictor of post-operative seizure occurrence, longer overall hospital stay, and disability in elderly liver recipients. In our elderly cohort, as already known from previous reports [10,14], HE is a predictor of NCs after liver transplantation. Other liver disease complications (ascites and/or variceal bleeding) or liver disease severity (MELD score) were associated with adverse post-operative outcomes, as extensively reported in the Results section. It is, finally, not surprising that a preoperative renal impairment, lower BMI, and perioperative sodium shift were associated with adverse outcome as toxic-metabolic encephalopathy and CPM.

Although we recognize the limited statistical power of our study, we consider it original in its exploration of whether the cSVD diagnosis before a liver transplant in elderly patients acts as a vulnerable background for acute postoperative adverse neurological outcomes. No other study has investigated, to the best of our knowledge, a crucial topic that could influence in the future the choice to include a neurological screening as a standard in all centers when an elderly cirrhotic patient is evaluated for a transplant; quite the opposite, it is likewise important to establish that this screening is of no use. According to our results, albeit not conclusive and only exploratory, a more severe chronic cerebrovascular disease increases the hospital stay (overall and in ICU) and increases the risk of postoperative seizures. The vascular load may work as an additional predisposing factor for lowering the seizure threshold when other risk factors occur peri-operatively, with metabolic derangements and drug toxicity being the most common.

NCs after liver transplantation are extensively reported as life-threatening conditions, highly associated with mortality, morbidity, loss of functional autonomy, and increased healthcare costs. Therefore, there is a need to persist in attempts to identify those modifiable risk factors in order to carry out a more precise pre-operative risk stratification of patients before surgery. Exploration of risk factors in elderly liver recipients is still a non-standardized clinical practice, and hence we would like to encourage setting up a pre-operative neurological screening for liver transplant candidates over the age of 65 in all transplantation centers, mainly looking at cSVD and its severity (lesion load) as a vulnerable background for acute NCs or non-NCs. A multi-center prospective study design would allow clinicians to enroll a larger number of patients for a more powerful statistical analysis. Even if increased over time, the total number of elderly individuals transplanted in each center will remain limited; thus, the involvement of multiple centers seems fundamental in reaching an appropriate sample size in a reasonable time for an urgent topic. Beginning a few years ago, at our transplantation institute, a brain MRI assessment is included as a routine in the liver transplantation eligibility workup for over-65-year-old liver disease patients. Therefore, we are confident that we can enroll a larger number of patients in the near future for a larger single-center study, which may help for a sample size estimation in preparation for a multi-center study.

## 5. Conclusions

We present the preliminary data, after an exploratory analysis of a small sample size, from a study looking at preoperative cSVD as a risk factor of adverse neurological and non-neurological outcomes after a liver transplantation in elderly patients. Although our experience is limited by the low number of cases and its retrospective nature, we hope to lay the groundwork for a larger innovative study that may led to a relevant conclusion with two possible scenarios: (a) cSVD is a risk factor for acute NCs after orthotopic liver transplantation in elderly recipients: identification of those patients at risk for the development of adverse outcome will better inform the patient and surgeon prior to undergoing the transplant. Patients at high risk for the development of NCs could be placed on a more aggressive or more targeted rehabilitation program. (b) cSVD is not a risk factor for acute NCs after liver transplantation in elderly recipients, thus suggesting that a preoperative brain MRI screening is little or no use and is time- and money-consuming. At this point, we address our exploration of risk factors for different conditions, because prevention of neurologic deficits remains an important goal for a successful liver transplant.

## Figures and Tables

**Table 1:** Patients’ baseline demographic and clinical characteristics.

Demographics	
Age years, median (IQR)	67 (67–68)
Gender (M/F)	15/7 (68%/32%)
BMI (Kg/m^2^), median (IQR)	25.53 (23.39–27.69)
Waiting list time, days	149.45 ± 350.90
Liver disease diagnosis, n (%)	
Alcoholic liver disease	1 (4.55%)
HCV-related HCC	8 (36.4%)
HCV-related cirrhosis	2 (9.09%)
HBV-related HCC	2 (9.09%)
NASH-related HCC	2 (9.09%)
NASH-related cirrhosis	2 (9.09%)
Cryptogenic cirrhosis	1 (4.55%)
Cryptogenic HCC	1 (4.55%)
Autoimmune HCC	2 (9.09%)
Epithelioid hemangioendothelioma HCC	1 (4.55%)
Severity of liver disease	
MELD score *, median (IQR)	11 (10–18)
UNOS status (2B/3)	6/16 (27%/73%)
Liver disease complications, n (%)	
Hepatic encephalopathy	10 (45,45%)
Ascites	11 (50%)
Variceal bleeding	16 (72.73%)
Abdominal procedures, n (%)	
TIPS	4 (18.18%)
Comorbidities, n (%)	
Diabetes mellitus	7 (31.82%)
Hypertension	7 (31.82%)
Atrial fibrillation	0
TIA	2 (9.09%)
Ischemic stroke	2 (9.09%)
Hyperlipidemia	1 (4.55%)
Renal failure	2 (9.09%)
Tobacco use, n (%)	
Never	10 (45.45%)
Previous	9 (40.91%)
Current	3 (13.64)
Cerebral small vessel disease, n (%)	
Fazekas score 0	0
Fazekas score 1	11 (50%)
Fazekas score 2	8 (36.36%)
Fazekas score 3	3 (13.64%)
Cognitive assessment	
MMSE *, median (IQR)	28 (27–30)
Donor variables	
Age years, median (IQR)	59.5 (52–71)
Gender (M/F)	10/12 (45%/55%)
Intraoperative variables	
Operation time (min) **, median (IQR)	352 (320–405)
Cold ischemia time (min) *, median (IQR)	395 (349–485)
Warm ischemia time (min) ***, median (IQR)	45 (39.5–56.5)
Peri-operative variables	
Sodium shift, n (%) ^†^	8 (36.36%)
Tacrolimus level POD 7 (ng/mL), median (IQR)	7.80 (6.90–8.90)
Tacrolimus mean level in POD-1-7 (ng/mL), median (IQR)	6.76 (4.93–8)
Magnesium level POD 7 (mEq/L), median (IQR)	1.90 (1.70–2.20)
Sodium level POD 7 (mEq/L), median (IQR)	140 (137–141)

IQR = interquartile range; HCV: hepatitis C virus; HCC: hepatocellular carcinoma; HBV: hepatitis B virus; NASH: nonalcoholic steatosis hepatitis; MELD: Model for End-stage Liver Disease; UNOS: United Network for Organ Sharing; TIPS: transjugular intrahepatic portosystemic stent; TIA: transient ischemic attack; MMSE: Mini Mental State Examination; POD: postoperative day; ng/mL: nanograms per milliliter; mEq/L: milliequivalents per liter; * analyzed in 15/22 patients; ** analyzed in 21/22 patients; *** analyzed in 16/22 patients; ^†^ we considered only sodium shift greater than 8 mEq/l in 24 h during the first week after liver transplantation.

**Table 2:** Peri-operative acute neurological complications.

Neurological Complication	n (Out of 22)	%
Toxic-metabolic encephalopathy *	3	13.64
Seizures *	1	4.55
Ischemic stroke/TIA	0	0
Intracranial bleeding	0	0
SNC infections	0	0
Central pontine myelinolysis *	2	9.09
Peripheral nervous system disease	0	0

The most frequent postoperative NC was toxic-metabolic encephalopathy, followed by CPM and seizures. * In 2/22 cases, we experienced an overlapping diagnosis: toxic metabolic encephalopathy and CPM in one case simultaneously with pharmacological-resistant seizures in another case.

**Table 3:** Peri-operative non-neurological complications.

Post-Operative General Complications	n (of 22)	%
Respiratory complications	9	40.91
Acute renal impairment with dialysis	4	18.18
Rejection	3	13.64
Delayed graft function (graft failure)	1	4.45
Infections	14	63.64

A total of 16/22 (72.7%) patients experienced at least one non-NC, with infections being the most common, followed by respiratory complications, acute renal impairment, rejection, and graft failure.

**Table 4:** Comparison of pre-operative risk factor profile in patients with post-operative acute NCs and patients without NCs.

Variables	noNCs	NCs	*p*-Value
Demographics			
Age years, median (IQR)	67.5 (67–69)	67 (66–67.5)	0.2295
Gender (M/F)	13/5 (87%/71%)	2/2 (13%/29%)	0.3881
BMI (Kg/m^2^), median (IQR)	25.53 (23.89–27.69)	24.87 (21.66–28.37)	0.7017
Waiting list time, median (IQR)	29 (15–89)	20.50 (9.50–57)	0.4691
Liver disease diagnosis, n (%)			
Alcoholic liver disease	1 (6%)	0 (0%)	0.4793
HCV-related HCC	6 (33%)	2 (50%)	
HCV-related cirrhosis	2 (11%)	0 (0%)	
HBV-related HCC	2 (11%)	0 (0%)	
NASH-related HCC	2 (11%)	0 (0%)	
NASH-related cirrhosis	1 (6%)	1 (25%)	
Cryptogenic cirrhosis	1 (6%)	0 (0%)	
Cryptogenic HCC	0 (0%)	1 (25%)	
Autoimmune HCC	2 (11%)	0 (0%)	
Epithelioid hemangioendothelioma HCC	1 (6%)	0 (0%)	
Severity of liver disease			
MELD score *, median (IQR)	11 (8–16)	14.5 (10.5–27)	0.2897
UNOS Status (2B/3)	5/13 (28%/72%)	1/3 (25%/75%)	0.9102
Liver disease complications, n (%)			
Hepatic encephalopathy	6 (33%)	4 (100%)	0.0154
Ascites	7 (39%)	4 (100%)	0.0270
Variceal bleeding	12 (67%)	4 (100%)	0.1757
Abdominal procedures, n (%)			
TIPS	3 (17%)	1 (25%)	0.6959
Comorbidities, n (%)			
Diabetes mellitus	6 (33%)	1 (25%)	0.7462
Hypertension	6 (33%)	1 (25%)	0.7462
Atrial fibrillation	0	0	
TIA	2 (11%)	0 (0%)	0.4844
Ischemic stroke	2 (11%)	0 (0%)	0.4844
Hyperlipidemia	1 (6%)	0 (0%)	0.6295
Renal failure	1 (6%)	1 (25%)	0.2211
Tobacco use, n (%)			
Never	8 (44%)	2 (50%)	0.6745
Previous	7 (39%)	2 (50%)	
Current	3 (17%)	0 (0%)	
Cerebral small vessels disease, n (%)			
Fazekas score 0	0	0	0.5158
Fazekas score 1	10 (56%)	1 (25%)	
Fazekas score 2	6 (33%)	2 (50%)	
Fazekas score 3	2 (11%)	1 (25%)	
Cognitive assessment			
MMSE *, median (IQR)	28 (27–30)	29 (18–30)	1.000
Donor variables			
Age years, median (IQR)	60.5 (51–73)	57.5 (54.5–62)	0.7015
Gender (M/F)	8/10 (44%/56%)	2/2 (50%/50%)	0.8400
Intraoperative variables			
Operation time (min) ^*^, median (IQR)	346 (290–405)	372 (334–475)	0.4505
Cold ischemia time (min) **, median (IQR)	378.5 (341.5–442)	405 (378–501)	0.3481
Warm ischemia time (min) ***, median (IQR)	45 (40–57)	50 (32–54)	0.7876
Peri-operative variables			
Sodium shift, n (%) ^†^	5 (28%)	3 (75%)	0.0758
Tacrolimus level POD 7 (ng/mL), median (IQR)	8.2 (6.9–9.3)	7.65 (5.8–7.9)	0.4692
Tacrolimus mean level in POD-1-7 (ng/mL), median (IQR)	6.83 (5.41–8.17)	5.32 (3.49–7.13)	0.2503
Magnesium level POD 7 (mEq/L), median (IQR)	1.9 (1.7–2.1)	2.15 (1.75–2.45)	0.4418
Sodium level POD 7 (mEq/L), median (IQR)	140 (137–141)	137 (132–143.5)	0.4922

HE and ascites predict a higher risk of NCs after OLTx in elderly recipients. * Analyzed in 21/22 patients; ** Analyzed in 15/22 patients; *** analyzed in 16/22 patients; ^†^ we considered only sodium shift greater than 8 mEq/l in 24 h during the first week after liver transplant.

**Table 5:** Neurological and Non-Neurological Complications distribution in different Fazekas scale groups.

	Fazekas 1	Fazekas 2	Fazekas 3	*p*-Value
NCs				
Toxic- metabolic encephalopathy	1 (9.1%)	2 (25%)	0	0.4621
Seizures	0	0	1 (33.3%)	0.0362
Neurotoxicity	1 (9.1%)	0	1 (33.3%)	0.2307
Central pontine myelinolysis	1 (9.1%)	0	1 (33.3%)	0.2307
Non-NCs				
Post-operative infections	5 (45.5%)	6 (75%)	3 (100%)	0.1547
Graft rejection	1 (9.1%)	1 (12.5%)	1 (33.3%)	0.5515
Acute renal impairment with dialysis	1 (9.1%)	3 (37.5%)	0	0.1935
Respiratory complications	5 (45.5%)	3 (37.5%)	1 (33.3%)	0.9032
Total	11	8	3	

The table shows a statistically significant association between Fazekas 3 score and seizures (*p* = 0.0362).

## Data Availability

The data presented in this study are available on request from the corresponding author. The data are not publicly available due to privacy protection rules.

## Abbreviations

cSVD: cerebral small vessels disease; WML: white matter lesions; IRRB: Institutional Research Review Board; EC: Ethics Committee; MRI: magnetic resonance imaging; MELD: Model for End-stage Liver Disease; POD: postoperative day; FLAIR: fluid-attenuated inversion recovery; GRE: gradient-echo; SWI: susceptibility weighted imaging; BMI: body mass index; TIA: transient ischemic attack; UNOS: United Network for Organ Sharing; TIPS: transjugular intrahepatic portosystemic shunt; NCs: neurological complications; LOS: length of stay; ICU: intensive care unit; MMSE: Mini Mental State Examination; IQR: interquartile range; HE: hepatic encephalopathy.

## References

[1] Lipshutz G.S., Busuttil R.W. (2007). Liver transplantation in those of advancing age: The case for transplantation. Liver Transplant..

[2] Stieber A.C., Gordon R.D., Todo S., Tzakis A.G., Fung J.J., Casavilla A., Selby R.R., Mieles L., Reyes J., Starzl T.E. (1991). Liver transplantation in patients over sixty years of age. Transplantation.

[3] Zetterman R.K., Belle S.H., Hoofnagle J.H., Lawlor S., Wei Y., Everhart J., Wiesner R.H., Lake J.R. (1998). Age and liver transplantation: A report of the Liver Transplantation Database. Transplantation.

[4] Keswani R.N., Ahmed A., Keeffe E.B. (2004). Older age and liver transplantation: A review. Liver Transplant..

[5] Wardlaw J.M., Smith C., Dichgans M. (2013). Mechanisms of sporadic cerebral small vessel disease: Insights from neuroimaging. Lancet Neurol..

[6] Schmidtke K., Hüll M. (2005). Cerebral small vessel disease: How does it progress?. J. Neurol. Sci..

[7] Ter Telgte A., van Leijsen E.M.C., Wiegertjes K., Klijn C.J.M., Tuladhar A.M., de Leeuw F.E. (2018). Cerebral small vessel disease: From a focal to a global perspective. Nat. Rev. Neurol..

[8] Guarino M., Benito-Leon J., Decruyenaere J., Schmutzhard E., Weissenborn K., Stracciari A., EFNS (2006). EFNS guidelines on management of neurological problems in liver transplantation. Eur. J. Neurol..

[9] Ferguson K.J., Cvoro V., MacLullich A.M.J., Shenkin S.D., Sandercock P.A.G., Sakka E., Wardlaw J.M. (2018). Visual Rating Scales of White Matter Hyperintensities and Atrophy: Comparison of Computed Tomography and Magnetic Resonance Imaging. J. Stroke Cerebrovasc. Dis..

[10] Wu S.Y., Chen T.W., Feng A.C., Fan H.L., Hsieh C.B., Chung K.P. (2016). Comprehensive risk assessment for early neurologic complications after liver transplantation. World J. Gastroenterol..

[11] Vizzini G., Asaro M., Miraglia R., Gruttadauria S., Filì D., D’Antoni A., Petridis I., Marrone G., Pagano D., Gridelli B. (2011). Changing picture of central nervous system complications in liver transplant recipients. Liver Transplant..

[12] Lin R., Svensson L., Gupta R., Lytle B., Krieger D. (2007). Chronic ischemic cerebral white matter disease is a risk factor for nonfocal neurologic injury after total aortic arch replacement. J. Thorac. Cardiovasc. Surg..

[13] Zivković S.A. (2013). Neurologic complications after liver transplantation. World J. Hepatol..

[14] Fu K.A., DiNorcia J., Sher L., Velani S.A., Akhtar S., Kalayjian L.A., Sanossian N. (2014). Predictive factors of neurological complications and one-month mortality after liver transplantation. Front. Neurol..

